# Endothelial Dysfunction in Diabetes Is Aggravated by Glycated Lipoproteins; Novel Molecular Therapies

**DOI:** 10.3390/biomedicines9010018

**Published:** 2020-12-27

**Authors:** Laura Toma, Camelia Sorina Stancu, Anca Volumnia Sima

**Affiliations:** Lipidomics Department, Institute of Cellular Biology and Pathology “Nicolae Simionescu” of the Romanian Academy, 8, B.P. Hasdeu Street, 050568 Bucharest, Romania; laura.toma@icbp.ro (L.T.); camelia.stancu@icbp.ro (C.S.S.)

**Keywords:** diabetes, hyperglycemia, glycated lipoproteins, glycated LDL, glycated HDL, endothelial cell dysfunction, molecular mechanisms, epigenetic factors, therapeutic approaches

## Abstract

Diabetes and its vascular complications affect an increasing number of people. This disease of epidemic proportion nowadays involves abnormalities of large and small blood vessels, all commencing with alterations of the endothelial cell (EC) functions. Cardiovascular diseases are a major cause of death and disability among diabetic patients. In diabetes, EC dysfunction (ECD) is induced by the pathological increase of glucose and by the appearance of advanced glycation end products (AGE) attached to the plasma proteins, including lipoproteins. AGE proteins interact with their specific receptors on EC plasma membrane promoting activation of signaling pathways, resulting in decreased nitric oxide bioavailability, increased intracellular oxidative and inflammatory stress, causing dysfunction and finally apoptosis of EC. Irreversibly glycated lipoproteins (AGE-Lp) were proven to have an important role in accelerating atherosclerosis in diabetes. The aim of the present review is to present up-to-date information connecting hyperglycemia, ECD and two classes of glycated Lp, glycated low-density lipoproteins and glycated high-density lipoproteins, which contribute to the aggravation of diabetes complications. We will highlight the role of dyslipidemia, oxidative and inflammatory stress and epigenetic risk factors, along with the specific mechanisms connecting them, as well as the new promising therapies to alleviate ECD in diabetes.

## 1. Introduction

The prevalence of diabetes mellitus (DM) is rapidly increasing worldwide [1]. The decreased quality of life of diabetic patients and the social and economic burden of this disease emphasize the need to establish the causative mechanisms of DM that will finally allow the identification of new therapies to cure diabetes and its associated vascular complications. Cardiovascular diseases (CVD) are the clinical manifestations of atherosclerosis, which represents one of the main vascular threats of diabetes. Published data show that the risk of acute cardiovascular events (such as stroke or myocardial infarction) is seven to ten times higher in diabetic patients compared to non-diabetic subjects [2]. In addition, microvascular afflictions, including retinopathy, nephropathy, neuropathy and limb ischemia, occur at a very high rate in diabetic patients compared to non-diabetic individuals [2].

The primary cause of the pathophysiologic alterations of the diabetic patient’s vasculature is the exposure to high levels of blood glucose. It is well known that high glucose (HG) can induce vascular complications in diabetic patients by affecting the normal function of the vessel wall’s cells. Unfortunately, large-scale clinical studies have shown that despite good glycemic control, the vascular complications persist and even evolve [3,4]. This phenomenon is known as the “metabolic memory” of the cells [5]. The first cells of the vessel wall exposed to plasma HG are the endothelial cells (EC). Constant plasma hyperglycemia or intermittent HG due to poor glycemic control induces EC dysfunction (ECD) [6,7,8]. ECD is considered a critical step in the initiation and evolution of atherosclerosis [9]. It favors an increased trans-endothelial transport of plasma proteins and lipoproteins (Lp), stimulates the adhesion and sub-endothelial transmigration of blood monocytes, supports the migration and proliferation of vascular smooth muscle cells (SMC) from the media to the intima and impedes the fibrinolytic processes, finally increasing the risk of cardiovascular events in diabetic patients [10]. Prolonged plasma HG induces also the formation of advanced glycation end products (AGEs), which by non-enzymatic attachment to proteins compromise their proper functioning. The interaction between the receptor for AGE (RAGE) and AGE proteins activates numerous signaling pathways and represents a powerful determinant of ECD [11]. Glycated lipoproteins (gLp), which are formed in excess in the plasma of diabetic patients, are ligands for RAGE and contribute substantially to ECD.

The aim of the present review is to select and summarize the molecular mechanisms that determine ECD due to cells’ interaction with gLp and to present new therapeutic strategies to alleviate CVD in diabetes. Special attention is given to the interaction of EC with glycated low-density lipoproteins (gLDL) and glycated high-density lipoproteins (gHDL) as important players in the accelerated-atherosclerotic process in diabetes.

## 2. Structural and Biochemical Alterations of Proteins and Lp Induced by High Glucose

### 2.1. Generation of Advanced Glycation End Products

Diabetes is a metabolic disorder affecting people worldwide, its major complication being vascular diseases, especially accelerated atherosclerosis. Characteristic of diabetes is the increased levels of blood glucose. Hyperglycemia induces the non-enzymatic glycation of blood proteins, resulting in AGE formation [12]. Extracellular glycation of different molecules can be the result of Maillard reactions or of prolonged oxidative stress. In Maillard reactions, AGEs formation starts with the condensing reaction between the carbonyl group from glucose (or other reducing sugars) and the primary amino groups of proteins, lipoproteins, nucleic acids or other molecules. The reversible Schiff bases formed are transformed into Amadori products and then into various irreversible, crosslinked, fluorescent and chemically reactive adducts [13]. The rearrangement of Amadori products to form AGEs occurs via oxidative or non-oxidative processes. The rearrangement of Schiff bases takes place at an alkaline pH, while the Amadori rearrangements start at a low pH, having a lower reaction rate [14].

Another source of AGEs is the advanced lipoxidation end products (ALEs) that result from successive oxidation cascades. The formation of ALEs starts with the peroxidation of lipids (such as polyunsaturated fatty acids from cellular membranes), leading to reactive carbonyl species (RCS), such as malondialdehyde (MDA) or 4-hydroxy-trans-2-nonenal (4-HNE), or to α-oxoaldehydes, such as glyoxal, methylglyoxal, 3-deoxyglucuson and acrolein [15]. After further arrangements, RCS can generate AGEs such as Nε-carboxyethyllysine (CEL), arginine pyrimidine, pentosidine, pyrralin, glyoxal lysine and Nε-carboxymethyllysine (CML) [14].

Although protein glycation takes place in vivo in tissues and fluids even under physiological conditions, in diabetes, this reaction takes place at a faster rate because of the increased availability of glucose and its catabolic aldehydes, such as glycoaldehyde and methylglyoxal and increased oxidative stress [16,17]. Most of the time, the AGEs and ALEs coexist in vivo, acting together in macromolecular complexes, such as Lp, and contributing to the aggravation of the vascular damage in diabetes.

### 2.2. Irreversible Glycation of Lipoproteins

The formation of AGEs represents an important mechanism in the propagation of the atherosclerotic process in diabetes. AGEs accumulate on long-lived proteins, affecting the cellular membranes, cytosolic proteins, the components of the basal lamina and of the extracellular matrix [18]. Plasma Lp can also be modified by excessive glucose, resulting in non-enzymatically glycated Lp (AGE-Lp).

Two classes of Lp, the low-density Lp (LDL) with pro-atherogenic potential and the protective, anti-atherogenic high-density Lp (HDL), are mainly involved in the atherosclerotic process. LDLs are the main cholesterol carriers in human plasma and, in pathological conditions, LDLs accumulate in the subendothelium as extracellular modified Lp, contributing to the atherosclerotic process by releasing pro-inflammatory bioactive lipids (such as oxidized phospholipids), inducing immune responses (by promoting the recruitment of immuno-inflammatory cells, such as monocytes, neutrophils, lymphocytes, or dendritic cells) or by being taken up by macrophages and determining the formation of foam cells [19]. Apart from LDL, lipoprotein (a) (Lp(a)) has received increasing interest in recent years and is now recognized as an important independent risk factor for CVD in patients with or without type 2 DM (T2DM) [20]. Lp(a) is composed of an LDL-like particle that binds to apoprotein(a) (apo(a)) via plasminogen-like domains, has structural similarity to plasminogen and tissue plasminogen activator (t-PA) and thereby may induce diminished fibrinolysis and even thrombogenesis due to the stimulation of the secretion of plasminogen activator inhibitor-1 (PAI-1). Further, Lp(a) carries cholesterol and binds atherogenic proinflammatory oxidized phospholipids, thus impairing the endothelial proper function and stimulating the attraction of inflammatory cells [21]. HDL plays important roles in maintaining the homeostasis of the vascular system, mainly by exerting antioxidant and anti-inflammatory effects and by participating in the reverse cholesterol transport process [19].

**Figure 1:**
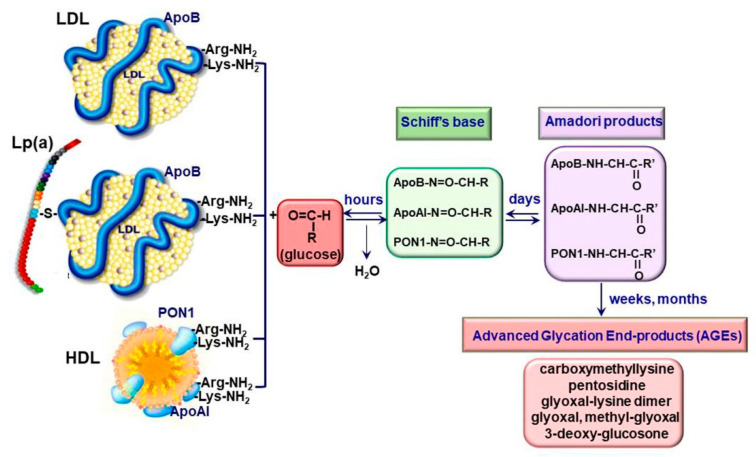
Formation of irreversibly glycated lipoproteins by Maillard reactions.

In LDL, glycosylation affects the apolipoprotein B (apoB), the main protein of this class of Lp. The irreversible glycation of native LDL (nLDL) starts with the non-enzymatic addition of reducing sugars to the positively charged arginine and/or lysine residues of apoB and continues with the formation of sugar-amino acid adducts, collectively known as AGE (Figure 1).

Since the arginine and lysine residues from apoB are important for the specific recognition by the LDL receptor (LDLR), an impaired LDLR-mediated uptake, a decreased gLDL clearance and an increased gLDL mean lifetime in the plasma of diabetic patients occur [16,22]. Furthermore, gLDL has an increased susceptibility towards oxidative modification, a critical step in atherogenesis [17]. It was reported that irreversible glycated LDL (AGE-LDL) has a 5-fold increased mean level of lipid peroxides, a reduction of the free amino groups of apoB and a higher gel electrophoresis mobility, reflecting the increase in the overall negative charge and the loss of positive charges [23]. Later, Deleanu et al. showed that AGE-LDL has an increased content of conjugated dienes, 4-HNE, MDA and 7-ketocholesterol compared to nLDL. In addition, the composition in free fatty acids is different in AGE-LDL compared to nLDL, with a decrease of linoleic acid, dihomogammalinolenic acid and arachidonic acid content being observed [24].

The levels of circulating Lp(a) are increased in T2DM and type 1 DM (T1DM) patients and positively correlated with blood concentrations of glycated hemoglobulin A1c. In addition, the levels of glycated Lp(a) are elevated in diabetic patients [25].

In the plasma of diabetic subjects, HDL main proteins become glycated; thus HDL lose their protective function. Godfrey et al. showed that apoAI, the main protein of HDL, is glycated in HDL isolated from diabetic patients. The in vitro glycation of apoAI appears at sites involved in membrane fusion and ligand binding and induces structural alterations of HDL particles. These modifications determine the decrease in HDL stability and functionality in plasma [26]. Kashyap et al. reported that the glycation of apoAI at Lys residues (Lys-12, Lys-96, Lys-133, and Lys-205) favors the appearance of apoAI crosslinking and is associated with a decreased apoAI half-life compared to its non-glycated form [27]. Proteomic analysis revealed changes in 7 of the 45 identified proteins in HDL from T2DM patients, including apolipoprotein (apo) A-II, apoE and PON-1. The data demonstrate early changes in the lipid and protein composition of specific HDL subspecies in adolescents with T2DM that are related to early markers of arterial disease [28].

### 2.3. Receptors for AGE-Proteins

The interaction of AGE-proteins with the cells is mediated by specific receptors. These include the type I cell surface receptor for AGEs (RAGE), AGE-receptor complexes (AGE-R1/OST-48, AGE-R2/80K-H, AGE-R3/galectin-3) and some members of the scavenger receptors family (SR-A; CD36, SR-BI, LOX-1; FEEL-1; FEEL-2) [14]. Unlike AGE-receptor complexes, which take up AGEs and participate in their clearance from circulation, RAGE interaction with AGEs leads to the activation of the intracellular signaling pathways, leading to increased oxidative and inflammatory stress [11,29]. RAGE belongs to the immunoglobulin (Ig) superfamily and is expressed on various cell types: EC, SMC, monocytes/macrophages, T-lymphocytes, dendritic cells, fibroblasts, neuronal cells, glia cells, chondrocytes, keratinocytes. Besides AGE, RAGE recognizes a large number of ligands, including HMGB1, S100/calgranulin protein, amyloid β peptide and lipopolysaccharides (LPS) [13]. Essential for RAGE signaling is Diaphanous1 (DIAPH1)/mammalian diaphanous-related formin (mDia1), a protein involved in the cytoskeleton organization that interacts directly with the cytoplasmic domain of RAGE. Key down-effectors of DIAPH 1 include the activation of Src kinase, Rho GTP-ases (including cdc42 and Rac), glycogen synthase kinase3b (GSK3b), AKT and Rho-associated, coiled-coil-containing protein kinase (ROCK). Activation of these signaling pathways was linked with different pathological situations including myocardial ischemia, diabetes-associated nephropathy, retinopathy and inflammation [30,31]. Besides mDia1, it was reported that the RAGE cytoplasmic domain can interact also with ERK and the adaptor protein for toll-like receptors (TIRAP) [32,33].

Multiple alternative splice forms of RAGE with partial functionality were also discovered. Of them, three isoforms are most important: (1) the N-truncated RAGE lacking the extracellular V-domain, thus preventing AGE binding; (2) the dominant-negative RAGE that lacks an intracellular domain, remaining anchored to the cell surface and permitting AGE interaction, but without stimulating the intracellular signaling; and (3) the endogenous secreted RAGE (esRAGE) that lacks both the transmembrane region and intracellular domain. Besides these isoforms, another soluble form of RAGE is formed by the actions of MMP-9 and ADAM-10 matrix metalloproteinases that cleave the cytosolic domain of the RAGE protein [34]. Together with esRAGE, this form of truncated RAGE provides the soluble form of RAGE (sRAGE). Since sRAGE is lacking the transmembrane and cytoplasmic domains, the ligand binding to sRAGE is unable to trigger the intracellular signaling cascades. These features make sRAGE an anti-inflammatory, effective decoy and competitive inhibitor for full-length RAGE, being involved in the scavenging and clearance of AGEs from circulation [11]. The pathophysiological role of RAGE will be discussed in the next sections.

## 3. Endothelial Cell Dysfunction in Diabetes

ECs are instrumental for maintaining the homeostasis of the vascular system due to their multiple functions. It is known that ECs participate in the regulation of the vascular tone by secreting different vasodilators (such as nitric oxide, prostacyclin) and vasoconstrictors (endothelin, thromboxanes). They act as a selective barrier to control the exchange of macromolecules between the blood and tissues, control the extravasation and the traffic of pro-inflammatory leucocytes by regulating the expression of the cell adhesion molecules and cytokines and keep the balance between the pro-thrombotic and pro-fibrinolytic factors [35,36].

In diabetes, the primary metabolic modification is the chronically elevated blood glucose. ECs are the first cells of the vascular wall that interact with the blood-increased glycemia and suffer structural and functional alterations [18,35]. The structural modifications of EC in diabetes start with the switch to a secretory phenotype, as demonstrated by the overdevelopment of the rough endoplasmic reticulum (RER) and Golgi complexes, the enrichment of the intermediary filaments and Weibel–Palade bodies, the enlargement of the inter-endothelial junctions, and the increase in the number of plasmalemmal vesicles favoring the formation of transendothelial channels [37,38]. These modifications determine the formation of a hyperplasic basal lamina and the increase in EC permeability, favoring the subendothelial accumulation of native and modified LDLs, which contribute to atheroma formation [18].

**Figure 2:**
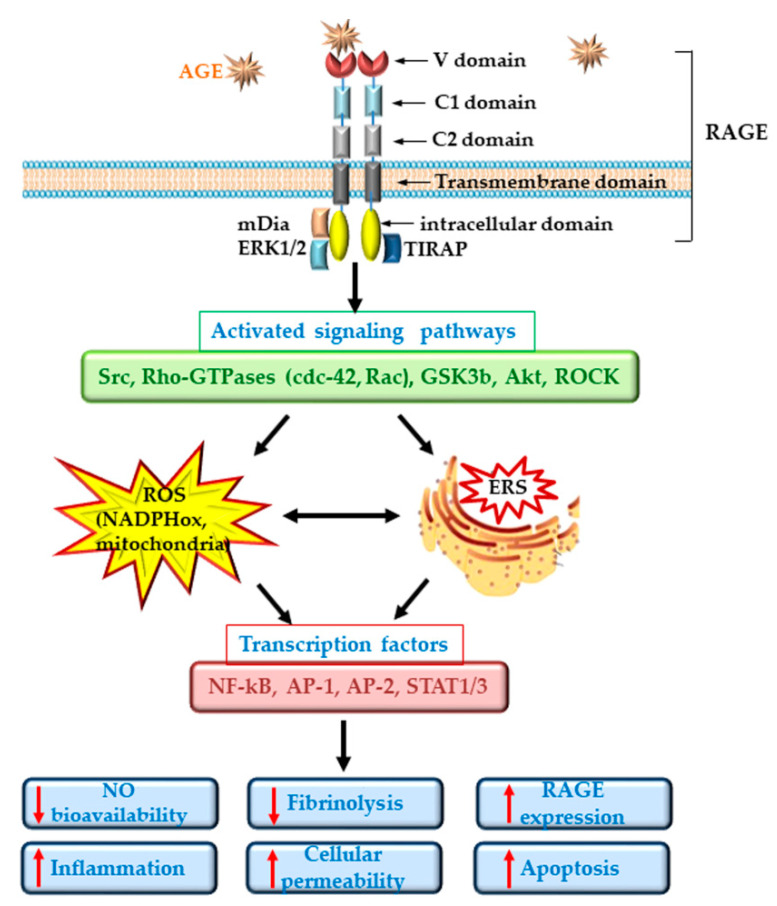
Schematic representation of advanced glycation end products (AGEs)/receptor for AGE (RAGE) interactions. The figure depicts the main signaling pathways, cellular processes and transcription factors involved in the generation of cellular dysfunction determined by AGEs. The black, thick arrows indicate the succesive activation of different signaling pathways and transcription factors stimulated by AGEs/RAGE interaction; the two headed black arrows indicate the interconnection between oxidative stress (ROS) and endoplasmic reticulum stress (ERS); the red arrows indicate the stimulation (up-headed arrows) or inhibition (down-headed arrows) of cellular processes which determine the dysfunction of endothelial cells.

ECD in diabetes is well documented and is regarded as an important player in the pathogenesis of CVD [39]. Dysfunctional ECs suffer a shift to a vasoconstrictor, pro-thrombotic and pro-inflammatory phenotypes. RAGE plays an important role in the development of the vascular complications associated with diabetes. It was demonstrated that deletion of RAGE in different animal models determines a significant attenuation of the atherosclerotic process [40,41]. The specific deletion of the cytoplasmic domain of RAGE of EC in transgenic mice was associated with a decrease in the inflammatory stress, revealing a prominent role for RAGE in ECD [40]. Studies in cultured EC have demonstrated that the interaction of different AGEs with RAGE determines the development of oxidative stress by activation of NADPH oxidase or induction of mitochondrial dysfunction [11,42,43] and lowers the bioavailability of nitric oxide (NO) [35]. In addition, series of intracellular phosphorylation reactions leading to the activation of MAPK (such as ERK1/2, p38), the enhancing of Jak/Stat signaling pathway and the activation of nuclear factor kappa B (NF-kB) are induced [14]. These effects result in the stimulation of the synthesis of pro-inflammatory cytokines and chemokines, including interleukin-6 (IL-6), monocyte chemoattractant protein 1 (MCP-1), tumor necrosis factor α (TNFα) and transforming growth factor β (TGF-β) and the overexpression of adhesion molecules, such as vascular cell adhesion molecule (VCAM-1) or intracellular cell adhesion molecule (ICAM-1), exacerbating the atherosclerotic process in diabetes [29,44,45]. Of great importance, it was demonstrated that AGEs increase the endothelial hyper-permeability by dissociating the adherens junctions through RAGE-mDia1 binding [46]. Interestingly, active NF-kB is also involved in the transcription of RAGE and of some of its ligands (such as HMGB1). Thus, the primary activation of RAGE unfortunately generates a positive feedback loop of self-sustained activation cycle, through NF-kB, amplifying the deleterious effects of AGE/RAGE interactions [47] (Figure 2).

In diabetic patients, the alteration of EC function was measured as: decreased forearm blood flow [48,49], increased levels of soluble adhesion molecules such as E-selectin, soluble VCAM-1 or soluble ICAM-1 [50,51], elevated plasma levels of von Willebrand factor (vWF) and PAI-1 [49,52,53]. More than being just a consequence of diabetes, ECD plays an important role in the development of microvascular (nephropathy, retinopathy, neuropathy) and macrovascular (ischemic heart disease, stroke, peripheral vascular disease) complications of diabetes [39].

## 4. Glycated Lipoproteins Detrimental Actions in Endothelial Cells

### 4.1. Reduction of Nitric Oxide Bioavailability

NO is essential in maintaining the functionality of the vascular system, due to its wide pleiotropic beneficial actions, having antiplatelet adhesion, anti-inflammatory, antioxidant and anti-apoptotic properties [35]. In diabetic conditions, the NO bioavailability is decreased due to the reduced endothelial NO synthesis or due to the interactions of NO with the excess pro-oxidant molecules (such as superoxide anion). In EC, NO is synthesized from L-arginine and O_2_ by the constitutive endothelial nitric oxide synthase (eNOS or NOS3) in a reaction that produces stoichiometric amounts of l-citrulline and NO. Besides L-arginine, the presence of cofactors such as heme, tetrahydrobiopterin (BH_4_), flavin adenine mononucleotide (FMN), flavin adenine dinucleotide (FAD) and NADPH is also required. ENOS functions as a homodimer, and its regulation is complex, involving transcriptional, post-transcriptional and post-translational mechanisms; any alteration of these mechanisms resulting in the decrease of NO production. Interaction with calmodulin, palmitoylation and myristoylation, phosphorylation by protein kinase B (Akt) or AMP-activated protein kinase (AMPK) at Ser1177 (Ser1179), and its localization in the cell’s membrane, determine eNOS activation. In contrast, the interactions with caveolin or the phosphorylation at Thr495/Tyr657 by the redox-active kinases (protein kinase C, PKC) promotes the inactivation of eNOS and the subsequent decrease of NO production [54]. The reactive oxygen species (ROS) can decrease NO synthesis through an interesting molecular mechanism: ROS lowers the levels of BH_4_ or L-arginine, and eNOS becomes “uncoupled”, generating superoxide instead of NO, thus exacerbating the oxidative stress [35]. In addition, the reaction between the superoxide molecule and NO determines the formation of peroxynitrites (ONOO-), highly reactive nitrogen species that exacerbate the oxidative stress and decrease the bioavailability of NO. An important participant in the peroxynitrite formation is the inducible NO synthase (iNOS) that can be stimulated by the interaction of AGE with RAGE [11]. Unlike eNOS, which synthesizes NO in small quantities over long periods of time, iNOS synthesizes large amounts of NO in short periods of time, thus stimulating the formation of peroxynitrites in a pro-oxidant medium and amplifying the oxidative stress [35,54].

**Figure 3:**
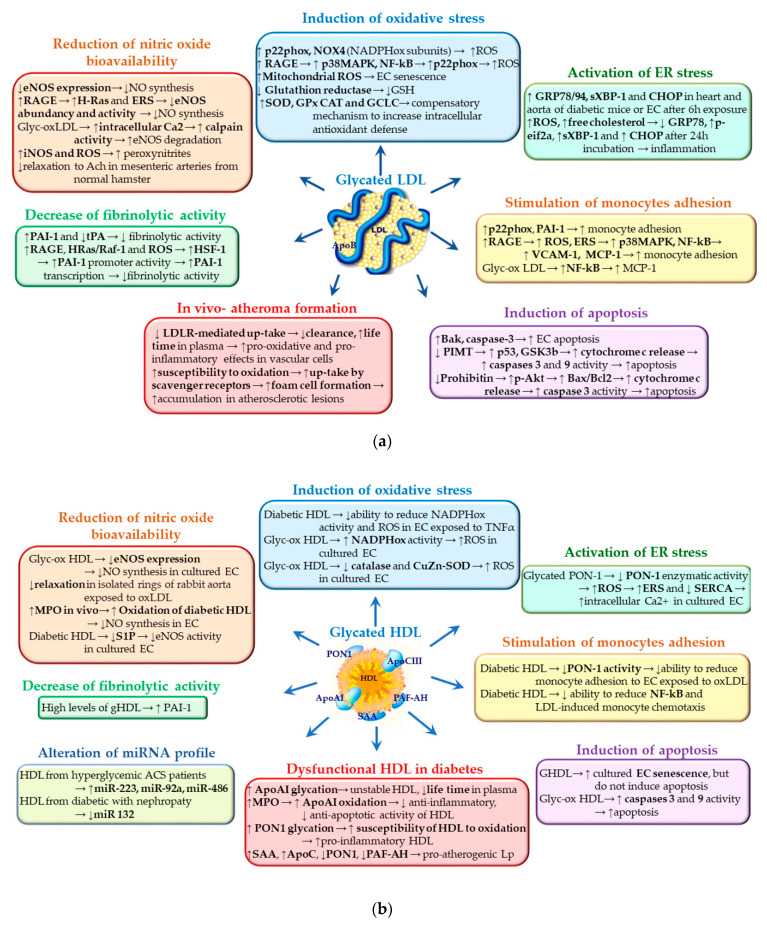
Molecular mechanisms of gLDL (**a**) or gHDL (**b**) contributing to endothelial cell dysfunction. The blue arrows indicate the detrimental actions of gLp in endothelial cells; the small black arrows indicate the specific molecular mechanisms by which gLp determine the dysfunction of endothelial cells as showed by experimental data.

The specific effects of gLp on NO synthesis and bioavailability (synthesized as shown in Figure 3a) were studied mainly in cultured ECs from various sources. Rabini et al. reported that the incubation for a short period of time of human aortic EC (HAEC) with LDL isolated from T1DM patients stimulates the production of NO in parallel with that of peroxynitrites [55]. Comparing the effects of gLDL obtained in vitro with those isolated from diabetic patients, Artwolh et al. demonstrated that both gLDLs determine the reduction of eNOS expression [56]. In agreement with this, Toma et al. showed that a 24 h incubation of cultured EC with 100 µg/mL AGE-LDL induces a slight inhibition of eNOS expression while increasing iNOS expression and ROS production [57]. Nair et al. confirmed and added to these results by showing that physiological concentrations of gLDL reduce the abundance of eNOS protein and its activity by a mechanism involving the stimulation of RAGE/H-Ras signaling pathway and of endoplasmic reticulum stress (ERS) [58]. The contribution of AGE-LDL to the alteration of the vascular reactivity was evidenced by using the myograph technique applied to mesenteric arteries from normal hamsters. Compared to nLDL, AGE-LDL decreased by 60% the relaxation of normal mesenteric arteries when stimulated with acetylcholine and by 40% when stimulated with sodium nitroprusside, demonstrating that AGE-LDLs affect both EC- and SMC-dependent relaxation of the arteries [59]. An interesting mechanism that might explain eNOS inhibition in cultured EC exposed to glycated and oxidized LDL (glyc-oxLDL) was described by Dong et al. [60]. They observed that the decrease in eNOS protein levels induced by glyc-oxLDL was accompanied by an increase in intracellular Ca(2+) levels, ROS production, and Ca(2+)-dependent calpain activity. By using specific pharmacologic inhibitors and silencing RNA, Dong et al. demonstrated that eNOS protein levels are decreased due to the activation of Ca(2+)-dependent calpain protease that upregulates the degradation of eNOS in EC exposed to glyc-oxLDL [60]. These studies prove without a doubt that gLDLs determine the decrease of NO bioavailability by affecting various molecular mechanisms, thus providing insights related to the development of cardiovascular complications in diabetes. The mechanisms by which gLDL induce ECD are summarized in Figure 3a.

It is known that one of the anti-atherosclerotic properties of native HDL is the stimulation of NO production in EC, determining the endothelium-dependent vasorelaxation [61]. Unfortunately, in diabetic patients, HDL is largely glycated [62]. The effects of gHDL on NO bioavailability (Figure 3b) were analyzed in a few studies. Matsunaga et al. demonstrated that 48h exposure of HAEC to 100 µg/mL glycated and oxidized HDL determines the decrease in eNOS expression and NO production [63]. Persegol et al. reported for the first time that HDL isolated from T2DM patients does not preserve the endothelium-dependent vasorelaxation function in isolated rings of rabbit aorta incubated with oxidized LDL [64]. In good agreement, in a randomized controlled trial, Sorrentino et al. showed that HDLs isolated from T2DM patients lose their ability to stimulate NO production from cultured HAEC and NO-mediated vasodilation of intact arterial segments. These effects were accompanied by an increase in gHDL peroxidation and HDL-associated myeloperoxidase (MPO) protein and activity, suggesting a mechanism in which MPO plays an important role [65]. More recently, it was shown that HDL isolated from T2DM loses its ability to stimulate eNOS activity and its capacity to suppress the NF-κB-mediated inflammatory response in TNFα-exposed EC. Interestingly, the loss of HDL’s ability to stimulate eNOS activity was correlated with a decrease of sphingosine-1-phosphate (S1P) levels in the plasma of T2DM; the authors suggested that the loss of S1P is a possible mechanism to explain the inability of HDL to exert its protective functions, contributing to the generation of vascular complications in diabetes [66].

The presented studies converge on the idea that gHDLs reduce NO bioavailability; however, more studies are needed for further clarification of the involved mechanisms. The mechanisms by which gHDLs induce ECD are summarized in Figure 3b.

### 4.2. Induction of Oxidative Stress

Under physiological conditions, ROS act as essential mediators of the redox signaling pathways involved in various cellular responses including proliferation, migration, differentiation or gene expression. In pathological situations, ROS is generated in excess, becoming toxic for cells, which leads to oxidation of molecules, enhancement of the inflammatory response, cellular aging or apoptosis [67]. Oxidative stress results from the imbalance between ROS production and ROS detoxification. In EC, the main sources of ROS are: the NADPH oxidases complex (specifically Nox2 and Nox4), the mitochondria and the uncoupled eNOS [35]. The cellular antioxidant defense system comprises a set of antioxidant enzymes, superoxide dismutase (SOD), catalase (CAT), glutathione peroxidase (GPx) and the thioredoxin system, which work together to decompose ROS, in particular the superoxide, to water and oxygen [67,68]. In EC, the antioxidant enzymes are stimulated by oxidative stress and participate actively in the adaptive response of the cells to ROS [69]. They have the ability to regenerate the proteins inactivated by oxidative stress and play an important role in EC survival by inhibiting the activation of c-Jun N-terminal kinase (JNK) and p38 mitogen-activated protein kinase (p38 MAPK) [67].

#### 4.2.1. Upregulation of the Main EC Pro-Oxidant Proteins by gLp

The induction of oxidative and inflammatory stress by gLp has been well documented, especially in the case of gLDL. Toma et al. reported that AGE-LDL stimulates NADPH oxidase activity by upregulating the p22phox and NOX-4 subunits and determines the increase of MCP-1 released in EC culture medium [70]. The upregulation of p22phox is p38 kinase and NF-kB-dependent, due to RAGE/AGE-LDL interaction. In addition, the authors showed that all these effects are significantly augmented in the presence of HG in the culture media. Interestingly, exposure of EC to nLDL in HG culture medium compared to normal glucose determines the stimulation of RAGE and NADPH subunits (p22phox, NOX4, and p67phox), supporting the concerted pathogenic potential of hyperglycemia and dyslipidemia in T2DM patients [70]. These results were confirmed by other groups, who reported that gLDLs increase the oxidative stress in cultured EC by stimulating the RAGE/NADPH oxidase axis [44,71]. Recent studies [44,45,72] show that stimulation of the oxidative stress was followed by the upregulation of the pro-thrombotic PAI-1 or of various pro-inflammatory proteins (C reactive protein, MCP-1, VCAM-1) that exacerbate the inflammatory state and stimulate monocyte adhesion to EC (Figure 3a).

Mitochondria play an important role in ROS generation, calcium homeostasis and cell survival. Increasing evidence demonstrates that functional alterations of mitochondria are involved in the promotion of ECD in diabetes [73]. Few studies focusing on the understanding of the effects of gLp on mitochondrial functioning have been done. It was reported that compared to nLDL, gLDL stimulates mitochondrial ROS generation in ECs [74,75]. ROS generation was accompanied by the attenuation of the activity of key enzymes from the mitochondrial electron transport chain, impaired mitochondrial oxygen consumption and the reduction of mitochondrial membrane potential [74]. These results were confirmed by a recent study [76] showing that the L5 subfraction of electronegative LDL augments mitochondrial free-radical production in cultured HAEC, leading to premature vascular endothelial senescence (Figure 3a).

HDLs isolated from diabetic patients, in contrast to HDL isolated from healthy subjects, lose their ability to reduce superoxide production and NADPH oxidase activity in TNFα-stimulated ECs [65]. Matsunaga et al. showed that oxidation of gHDL stimulates the increase of H_2_O_2_ by activating NADPH oxidase [63] (Figure 3b). Therefore, innovative therapies designed to increase endogenous antioxidants, in particular those associated with HDLs, will be valuable anti-diabetic treatment in addition to the existing ones.

#### 4.2.2. Modulation of the Activity of the Cellular Antioxidant Defense System by gLp

The cellular antioxidant defense system plays an important role in ROS detoxification, being critical in maintaining the cellular proper function. Studying the impact of gLp on the endothelial antioxidant system, Zhao et al. observed that both nLDLs and gLDLs induce stimulation of SOD, GPx and CAT activity after 24 h incubation with cultured ECs. Interestingly, the lowering of glutathione reductase, the enzyme responsible for the restoration of reduced glutathione (GSH) pool, was observed in gLDL-exposed ECs versus nLDL. In agreement with this result, the authors showed that GSH levels in EC are reduced by gLDLs [77]. Another antioxidant protein shown to be affected by gLDL was the catalytic subunit of glutamate cysteine ligase (GCLC), the first rate-limiting enzyme for GSH synthesis. Toma et al. reported an increase of GCLC gene expression in gLDL-exposed EC compared to cells exposed to nLDLs. Interestingly, using specific ERS inhibitors, the authors observed the decrease of GCLC levels to normal values. This result suggests that GCLC is increased as an attempt of the cells to restore their intracellular antioxidant protection by re-establishing the GSH pool consumed for the proper folding of proteins accumulated in the ER when the cells are exposed to gLDL [45].

The exposure of EC to glyc-oxHDL determined the down-regulation of catalase and Cu(2+), Zn(2+)-superoxide dismutase (CuZn-SOD) expression resulting in the formation of increased levels of H_2_O_2_ [63]. No specific studies regarding the effect of gHDL on ECs’ antioxidant defense have been published. The results are presented summarized in Figure 3a,b.

### 4.3. Activation of Endoplasmic Reticulum Stress

The ER is an organelle with multiple functions, involved in maintaining homeostasis, function and survival of the cells. The main role of the ER is the synthesis of proteins and lipids, the ER lumen being the place of residence for many foldases and chaperones (e.g., GRP78, GRP94) needed for proper protein folding. Due to its importance in cellular homeostasis, the ER functions are highly regulated. However, in pathological situations, in the lumen of the ER, a high amount of misfolded proteins may accumulate, inducing ERS [78]. To resolve ERS, cells initiate the unfolded protein response (UPR), a set of three signaling pathways meant to restore ER homeostasis, as follows: (1) the protein kinase-like ER kinase (PERK)/eukaryotic initiation factor (eIF)-2α branch to attenuate the novo translation of proteins; (2) the activating transcription factor (ATF)-6 upregulates the transcription of molecular chaperones needed to increase the capacity of ER folding; (3) the inositol-requiring enzyme 1 alpha (IRE1α)/spliced X-box binding protein 1 (sXBP-1) simulates the transcription of chaperones and activates the ER-associated protein degradation (ERAD) machinery. However, if ERS is prolonged and the cells do not restore the ER proper functions, the UPR generates the activation of the pro-inflammatory signaling cascades by the activation of NF-κB, p38MAPK and c-Jun N-terminal Kinase (JNK), finally leading to cellular apoptosis through the activation of CCAAT/enhancer-binding protein homologous protein (CHOP) and inhibition of anti-apoptotic Bcl-2, in parallel with the stimulation of the pro-apoptotic Bim [79].

It was reported that stressors such as high glucose, ROS or accumulated free cholesterol can be triggers of ERS in EC [80]. It is known that long-term dyslipidemia induces insulin resistance, a possible mechanism being the enhancement of ERS [81]. Although ERS was frequently associated with diabetes and vascular complications, the involvement of gLp in promoting ERS is not very well documented. Zhao et al. showed that the hearts and ascending aortae from diabetic mice present an increased level of UPR markers [82]. They showed that a 6 h incubation of cultured EC with gLDL stimulates ERS by the increase of GRP78/94, sXBP-1 and CHOP [82]. Toma et al. complemented these studies by demonstrating that a 24 h exposure of EC to gLDL increases ERS, probably due to an increase in accumulated free cholesterol in EC membranes induced by gLDL [45]. Unlike Zhao et al., Toma observed no modification of sXBP-1 levels, but a decrease in GRP78 levels. The dissimilarities might originate in the different exposure times to gLDL, being known that the IRE1α branch of UPR is attenuated after 8 h, despite the persistence of ERS [83]. Furthermore, the study demonstrated that ROS levels induced by gLDL were decreased by inhibitors of ERS and that ERS was alleviated by ROS inhibitors, showing an interconnection between ROS and ERS in gLDL-exposed HEC [45] (summarized in Figure 3a).

In normal conditions, native HDL exerts an anti-oxidant and anti-inflammatory action in EC activated by pro-inflammatory proteins or loaded with lipids. To study the effect of gHDL in EC, Yu et al. used a recombinant, non-enzymatically glycated PON-1. The glycated PON-1 presented a diminished enzymatic activity, and its incubation with ECs induced ERS and reduced the activity of sarco/ER Ca2+-ATPase (SERCA), thus increasing the intracellular levels of calcium by a mechanism involving the oxidative stress [84] (Figure 3b).

### 4.4. Stimulation of Monocytes Adhesion to Endothelial Cells

Monocytes’ adhesion and their subsequent transmigration into the subendothelium are instrumental for atherosclerosis inception and progression. The redox-sensitive NF-kB, the activator protein-1 (AP-1) and the redox-sensitive MAP kinases play important roles in the pathophysiology of diabetes, all being involved in the transcription of different pro-inflammatory cytokines, chemokines or cell adhesion molecules [85]. The overexpression of adhesion molecules (such as ICAM-1, VCAM-1, ninjurin-1) on EC plasma membrane stimulates monocytes adhesion and transmigration into the subendothelial space, contributing to atherosclerosis initiation and progression in diabetes.

Both gLDL and gHDL can activate ECs and stimulate the adhesion of monocytes to ECs through different molecular mechanisms [44,45,72]. Zhao et al. showed that incubation of cultured EC with gLDL determines an increased monocytes adhesion. Very interesting, they showed that the transfection of ECs with siRNA specific for p22phox or PAI-1 prevented the gLDL-induced monocytes adhesion to ECs, suggesting that the oxidative stress and the fibrinolytic modulators participate in the inflammatory processes in diabetes [44]. Toma et al. reported that monocytes adhesion is increased in ECs exposed to gLDLs due to the increase in VCAM-1 expression through signaling pathways involving RAGE, ROS, ERS stimulation and subsequent activation of p38MAP kinase and NF-kB [45]. Later, Toma et al. confirmed and added to the previous reports by evidencing the involvement of gLDLs in the secretion of MCP-1 and C reactive protein through mechanisms involving stimulation of RAGE, oxidative stress and ERS [72] (Figure 3a).

The effect of gHDLs on EC function was reported by Hedrick et al., who showed that gHDLs, compared to native HDLs, do not inhibit monocytes adhesion to HAEC exposed to oxidized LDL. The study concluded that the inability of gHDLs to exert anti-inflammatory protection is due to the significant reduction of PON-1 activity observed after HDL glycation [86]. HDLs isolated from diabetic patients did not inhibit the phosphorylation of NF-kB p65 subunit in TNFα-exposed EC compared to HDLs from normoglycemic subjects [66,87]. Moreover, HDL from T2DM patients had an impaired ability to inhibit LDL oxidation and LDL-induced monocyte chemotaxis [88] (Figure 3b). These studies demonstrate that the irreversible glycation of LDL and HDL participates substantially in the development of a pro-inflammatory state in EC, contributing to the major complications of diabetes.

### 4.5. Generation of Fibrinolytic Regulators

Intravascular thrombosis resulting from imbalances between the coagulation and the fibrinolytic processes plays a critical role in the diabetic vascular complications [89]. Plasmin, the main active product of the fibrinolytic system, maintains the fluidity of blood in the vasculature by breaking down the fibrin clots. Plasmin is generated from plasminogen through the balanced action of its activators, t-PA, urokinase plasminogen activators (u-PA) and the PAI-1, the physiological inhibitor of t-PA and u-PA. Vascular ECs synthesize both activators and inhibitors of fibrinolysis, playing a critical role in the homeostasis of fibrinolytic activity in the blood [89].

In diabetic patients, an attenuated fibrinolytic activity was determined [90,91], predisposing the patients to vascular accidents. Published data indicate that gLps have an important contribution to the alteration of the fibrinolytic system equilibrium in diabetes. Shen et al. extensively described the effects of gLps on the generation of fibrinolytic regulators from ECs. They were the first to demonstrate that gLDL amplifies the production of PAI-1 in parallel with the reduction of de novo synthesis of t-PA in HUVEC [92]. Similar results were obtained for LDL isolated from T1DM or T2DM patients or VLDL from T2DM patients [90]. Comparing the effects of oxidized LDL and gLDL on the fibrinolytic factors, Ma et al. showed that both modified Lp increase, PAI-promoter activity, PAI-1 mRNA level and its release from EC in a similar manner. Using specific inhibitors, the authors demonstrated that an intact Golgi apparatus is required for PAI-1 generation in ECs [93]. Zhao et al. reported that PAI-1 is upregulated by gLDL through a mechanism depending on the heat shock factor-1 (HSF-1) binding to PAI-1 promoter, which stimulates PAI-1 transcription [94]. Sangle showed later on that the endothelial RAGE, oxidative stress, and HRas/Raf-1 signaling pathways participate in the upregulation of HSF-1 or PAI-1 in EC exposed to gLDL [95] (summarized in Figure 3a).

Zhang et al. observed that glycation of Lp(a) enhances the production of PAI-1 and further decreases the generation of t-PA from HUVEC and human coronary artery EC [25]. Thus, glycation of Lp(a) attenuates the fibrinolytic activity in blood and contributes to the increased incidence of cardiovascular complications in diabetic patients with hyperlipoprotein(a).

Studies regarding the effects of gHDL on the fibrinolytic regulators expression in EC are scarce. Ren S et al. showed that although gHDLs do not significantly modify t-PA in EC, a high concentration of gHDLs (>/=100 µg/mL) moderately increases the release of PAI-1 (Figure 3b). However, it is interesting that HDL glycation does not affect the ability of HDL to reduce PAI-1 and to restore t-PA generation in ECs exposed to gLDL [91,96].

### 4.6. Induction of Endothelial Cell Apoptosis

Cellular apoptosis or programmed cell death is a physiological process important in the development of embryos and continuing throughout adult life. Apoptosis can be induced by two main separate pathways: one controlled by B-cell lymphoma 2 (BCL2), a family of proteins including anti-apoptotic BCL2 and bcl-XL and pro-apoptotic Bad, Bax, Bid or Bak, and the other one controlled by the so-called “death receptors”, including Fas, TNFα receptor 1 (TNFR1), death receptor 3 (DR3) or TNFα-related apoptosis-inducing ligand (TRAIL). Both these pathways converge to the activation of inactive initiator caspases (caspase-8 and -9) and the downstream effector caspases (e.g., caspases-3 and -7), finally determining the DNAse activation and cell’s death [97].

In diabetic conditions, an exacerbated apoptotic process was observed in ECs. Published data indicate hyperglycemia as the main causal factor for the development of EC apoptosis, a specific role being played by gLDL. Artwolh et al. demonstrated that 48 h exposure of HUVEC to 100 µg/mL of gLDLs isolated from diabetic patients determines the apoptosis of cultured ECs due to the increase in pro-apoptotic Bak and caspase-3 [56]. Similar results were obtained in ECs using the electronegative LDL subfraction L5 isolated from T2DM patients [98]. Li et al. showed that gLDLs decrease EC survival in a dose-dependent manner by promoting apoptosis [99]. The authors demonstrated that gLDLs increase p53 nuclear transcription factor levels and activate the glycogen synthase kinase 3 (GSK3b), determining the increase of the cytochrome c release from the mitochondria, which in turn successively activates caspase-9 and the effector caspase-3. Interestingly, Li et al. revealed that all these processes are abolished by the overexpression of Protein L-isoaspartyl methyltransferase (PIMT), a protein that plays a role in the repair and/or degradation of damaged proteins, whose levels are decreased by gLDL in an ERK1/2-dependent manner [99]. Another interesting mechanism by which gLDLs may induce EC apoptosis was described by Yin et al., who showed that HUVEC exposure to gLDL determines a significant increase in the Bax/Bcl-2 ratio, the release of cytochrome c from the mitochondria in the cytosol and stimulation of caspase-3 activity, determining cellular apoptosis [100]. In addition, the authors observed that gLDLs decrease the expression of prohibitin, a chaperone involved in the stabilization of mitochondrial proteins, involved in maintaining normal mitochondrial morphology and function. Prohibitin overexpression reversed the apoptotic process by inhibiting Akt phosphorylation, decreasing Bax/Bcl2 ratio, cytochrome c release and caspase 3 activity, showing that prohibitin plays a critical role in gLDL-induced EC apoptosis [100] (summarized in Figure 3a). In conclusion, the presented studies clearly demonstrate that gLDLs can and will stimulate the mitochondrial apoptotic pathways in EC.

The effect of gHDLs on EC apoptosis was also investigated. Matsunaga et al. showed that 100 µg/mL gHDL do not stimulate the apoptosis of HAEC. In contrast, if gHDL is additionally oxidized (glyc-oxHDL), a significant increase of caspase 3 activity and expression and caspase 9 expression, as well as HAEC apoptosis, are induced, suggesting that the oxidation process, rather than glycation, is detrimental for HDL function or pro-apoptotic effects [101]. Although HDL glycation is not sufficient to induce EC apoptosis, the glycation modifications suffered by gHDLs can determine EC senescence [102]. Park et al. reported that exposure of HUVECS to fructated ApoAI determines the increase in cellular senescence along with a lysosomal enlargement (Figure 3b).

## 5. Glycated Lipoproteins in Diabetes

### 5.1. Glycated LDL Participate in Atheroma Formation

Glycation determines the crosslinking of proteins that become more resistant to proteolysis and accumulate in different tissues, thus affecting their function [12]. In atheroma from diabetic patients, LDLs, AGE-proteins and oxidation products (such as 4-HNE-proteins) co-localize, being present intracellularly (in foam cells derived from macrophages or in SMC of the fibrous cap), as well as extracellularly, in the necrotic areas formed near the internal elastic lamina [103,104]. Tames et al. showed that glycated apoB levels are about two-fold higher in DM patients compared to healthy subjects, reaching a concentration of 9.3 mg/dl [105]. It was reported that LDL glycation determines structural alterations in Lp, inhibiting their recognition and uptake by the specific LDLR, allowing an enhanced uptake by scavenger receptors present in macrophages, promoting foam cells and atherosclerotic plaque formation [16]. Using an original model of hamsters with T1DM generated by injection with streptozotocin, and made hyperlipidemic by feeding a high-fat diet for 12 weeks, Simionescu et al. showed that levels of gLDL doubled in hamsters’ plasma compared to normal ones [106]. Using the model of streptozotocin-injected hamster with/without hyperlipidemia, Sima and Stancu showed that the aortic intima contains focal deposits of AGE-proteins and modified LDL, distributed either diffusely in the extracellular space or associated with SMC or macrophage-derived foam cells [104]. MDA-Lys and HNE-Lys adducts have been previously detected in atherosclerotic plaques of diabetic or diabetic-hyperlipidemic hamsters [107] or in atheroma from diabetic patients [104]. Using different methods to block the effects of reactive carbonyl species, it was reported that these compounds have robust pro-inflammatory properties, thus contributing to atherosclerosis development [108].

### 5.2. Dysfunctional HDL Are Pro-Atherogenic Particles in Diabetes

Functional HDLs have been reported to mediate different beneficial actions in normal physiological conditions by (i) participating in the glycemic control by interacting with pancreatic beta cells [109], (ii) inhibiting LDL glycation and oxidation [110], (iii) maintaining the function of EC through stimulation of NO production, (iv) reducing monocytes adhesion to EC and (v) participating in the reverse cholesterol transfer process [111]. Unfortunately, the metabolic disturbances characteristic for the diabetic conditions determine alterations in HDL composition and function, favoring the appearance of the dysfunctional HDL [112]. Dysfunctional HDLs, characterized by an increased content of MDA, MPO and ceruloplasmin, in parallel with decreased PON-1 protein and activity, were found in plasma from patients with coronary artery disease and/or diabetes and were demonstrated to play pro-inflammatory effects in EC [87].

In T2DM patients, Godfrey et al. observed an increase of methylglyoxal and dicarbonyl groups in ApoAI from HDL that was associated with the alteration of HDL functions [26]. HDL isolated from T2DM patients was shown to be dysfunctional, being unable to inhibit the inflammatory stress in TNFα-exposed EC [66] or LDL oxidation [88]. ApoA-I was demonstrated to also be a target of MPO, affecting the anti-inflammatory and anti-apoptotic activity of HDL in ECs or impairing the reverse cholesterol transport mediated by ABCA-1 [113,114]. Another protein associated with HDL and known to be affected in diabetes is the anti-oxidant enzyme PON-1. PON1 levels and activity were demonstrated to be low in T1DM or T2DM patients [115,116] determining an impaired antioxidant capacity of HDL [116,117]. Trying to decipher the mechanism of PON-1 regulation, Stancu et al. used the model of the hyperlipidemic hamster that in time develops hyperglycemia, similar to T2DM. These pathological conditions were associated with a decrease in PON-1 protein and activity in the plasma, small intestine and liver through a mechanism regulated by LXR and PPAR gamma [81]. Besides glycation and oxidation, other alterations in HDL composition were observed in diabetes. Proteomic studies showed that diabetic HDL is enriched in acute-phase reactant serum amyloid A (SAA) and apolipoprotein C-III, while the activity of PON-1 and platelet-activating factor-acetylhydrolase (PAF-AH) is decreased, causing the shift of HDL from anti-atherosclerotic to pro-atherogenic Lp [112,118]. HDL subclasses’ distribution was also altered in diabetes. Dullaart et al. reported that in the plasma of T2DM patients, large and medium-size HDL particles’ number decreased, while small HDL particles were increased [119]. In good agreement, epidemiological studies showed that HDL2, the larger, less dense subfraction of HDL, is negatively correlated with the development of T2DM [120].

In T1DM patients, HDLs are dysfunctional, presenting a low antioxidant protection [121] and a decreased capacity to participate in the reverse cholesterol transport [122]. In parallel, the increase was reported sof the pro-inflammatory SAA in HDL2 and HDL3 subfractions of HDL isolated from T1DM patients with poor glycemic control [123].

### 5.3. Hyperglycemia Alters miRNAs Profiles in Plasma and Lipoproteins

It is known that Lps are carriers for microRNAs (miRNAs) in plasma [124,125,126]. MiRNAs are small noncoding RNAs formed by approximately 22 nucleotides, demonstrated to regulate different target genes by post-transcriptional mechanisms. Using different experimental models, it was shown that miRNAs expression is modulated by diabetic conditions, being reported in the few existing studies to correlate with the presence of diabetic complications. Simionescu et al. demonstrated that circulating miR-125a-5p and miR-146a levels are positively correlated with hyperglycemia in the serum of coronary artery of affected patients [127]. In agreement with this, Huang et al. determined a 5-fold increase in miR-146a and miR-155 in the kidney samples obtained from patients with diabetic nephropathy and showed that these miRNAs induce inflammatory stress in glomerular EC in culture through the stimulation of TNFα secretion and NF-kB activation [128]. Published data indicate that miRNAs are mostly associated with HDL and can be transferred to cultured cells, thus affecting cells’ functionality by modulating their genes’ expression [124,125]. For instance, it was demonstrated that HDL transfers a functional miR-223 to EC, thereby downregulating the expression of ICAM-1, thus exerting an anti-inflammatory response [129]. In a study that evaluated miRNAs in the plasma and HDL from diabetic subjects with stable (SA) or unstable angina (ACS), Simionescu and Niculescu et al. demonstrated that miR-223, miR-92a and miR-486 were increased in HDL from hyperglycemic ACS patients compared to normoglycemic ones. Interesting, the distribution of these analyzed miRNAs in HDL was statistically different in the two groups of patients, discriminating between SA and ACS patients [130]. Later on, Florijn et al. demonstrated an altered distribution of miRNAs in HDL isolated from diabetic patients with nephropathy (DN), namely a decreased level of miR-132 was found in HDL from DN patients compared with healthy subjects. Trying to find the pathological relevance of these results, Florijn et al. tested the functional impact of HDL-associated miRNAs in ECs and demonstrated that HDL-miR-132 significantly stimulated the angiogenic capacity of ECs, suggesting that HDL enrichment with miR-132 represents a causal mechanism for the advancement of DN [131].

## 6. Promising Therapies to Reduce ECD in Diabetes

### 6.1. In Vitro Approaches to Decrease the Effects of gLp in EC

Considering the importance of EC in maintaining the homeostasis of the vascular system, the finding of therapeutic strategies to reverse ECD is of great importance.

The beneficial effects of some compounds (amlodipine, caffeic acid, cyanidin-3-glucoside, procyanidin B2) to restore the proper function of EC affected by gLp was evaluated in a few in vitro studies. It was shown that amlodipine, a calcium channel blocker, increases the bioavailability of NO in ECs exposed to gLDL, as demonstrated by the increase in the NO/peroxynitrite ratio in the culture media of the treated cells. This beneficial action was the result of the increased expression of eNOS, in parallel with the reduction in iNOS and NADPHox-dependent ROS induced by amlodipine. In addition, amlodipine reduced the activation of the pro-inflammatory NF-kB and p38 MAPK determining the decrease of monocyte adhesion to ECs, in part through downregulating MCP-1 and VCAM-1 expression [57]. Caffeic acid, a phenolic acid present in normal diets, was successfully tested for its antioxidant and anti-inflammatory effects in HECs exposed to gLDL. Caffeic acid reduced the secretion of CRP, VCAM-1, and MCP-1 in gLDL-exposed HEC by inhibiting RAGE expression and NADPHox-dependent ROS and attenuating the gLDL-activated ERS. In addition, it exerted anti-apoptotic actions, as demonstrated by CHOP inhibition and the restoration of the mitochondrial transmembrane potential [72]. Cyanidin-3-glucoside (C3G), an anthocyanin present in dark-skinned berries, was shown to reduce the intracellular superoxide production and to improve the viability of EC exposed to gLDL [71]. It was proven that the beneficial effects of C3G are the result of the downregulation of RAGE, thus determining the inhibition of NOX4 expression and normalization of mitochondrial-dependent ROS [71]. An interesting protective mechanism for EC function was described by Li X et al. for grape seed procyanidin B2 (GSPB2). They demonstrated that GSPB2 inhibits the apoptosis of ECs exposed to gLDL by increasing the expression of the protein PIMT, a repair enzyme that controls the release of cytochrome c and activation of caspase 3 and 9 [99]. Another mechanism by which GSPB2 reduced apoptosis, increasing the viability of ECs exposed to gLDL, was described later by Yin et al. [100]. In cultured ECs, the authors demonstrated that GSPB2 increases the expression of prohibitin, a protein implicated in cellular survival and apoptosis that is decreased by gLDL exposure. The increase in prohibitin by GSP2 reduced the cytochrome c release into the cellular cytosol, Bax/Bcl-2 ratio and attenuated the caspase-3 activity, thus protecting against apoptosis [100].

ERS decrease could be another approach for the diminution of gLp effects on ECs in diabetes. ERS inhibition by 4-phenylbutyric acid (PBA) effectively repressed the activation of eIF2α and CHOP signals and reversed AGEs-induced cell apoptosis in mesangial cells in vitro [132]. Stimulation of the ER folding capacity through chemical chaperones, such as tauro-urso-deoxycholate and PBA, promises to be a novel and valuable therapeutic attempt for preventing the effects of diabetes in the vascular cells [133].

### 6.2. Therapies Used to Alleviate the Vascular Disorders in Diabetes

In diabetic patients, several therapies have been used to reduce the vascular complications of this disease. These include changes in the lifestyle and/or administration of oral hypoglycemic drugs, vitamins, antioxidants, statins, etc., to target the pathological mechanisms that drive the development of diabetes and its complications. Unfortunately, although having beneficial properties for human health, some of them failed to reduce the major vascular complications in diabetes. For example, data from the large-scale Look AHEAD (Action for Health in Diabetes) trial showed that achieving changes of lifestyle and weight loss confers health benefits for DM patients (including decreasing sleep apnea, reducing the need for diabetes medication, maintaining physical mobility), but the cardiovascular risk remaine unchanged [134].

Since AGEs are important players in the progression of diabetic complications, the inhibition of the mechanisms that promote their formation is desirable. As highlighted, hyperglycemia and oxidative stress are the main contributors to AGE formation. The ability of different classes of therapeutic compounds with hypoglycemiant and anti-oxidant properties to ameliorate diabetes and its vascular complications was tested in different clinical studies and will be further presented.

#### 6.2.1. The Use of Hypoglycemiant Compounds

Oral hypoglycemiant compounds are an important class of drugs used in the treatment of diabetes. The compounds used at present act either by increasing the production of insulin (sulphonylureas, glinides and, more recently, incretin mimetics) or stimulating insulin sensitivity in the target tissue (insulin sensitizers such as metformin and thiazolidinediones) [134]. Metformin has been successfully used for long-term treatment as first-line therapy for T2DM to improve glycemic control in patients, being associated with a low risk of hypoglycemic events [135]. In a recent cohort study of USA veterans with T2DM, Wang et al. showed that metformin reduced the rate of CVD events [136]. In a meta-analysis, Lee et al. demonstrated that metformin is associated with fewer major adverse cardiac events in T2DM patients from Taiwan [137]. The beneficial cardio-metabolic effects of metformin were studied also in T1DM patients in a large double-blind randomized, placebo-controlled trial, the conclusion being that this drug reduces atherosclerosis progression [138]. In recent years, new antidiabetic drug classes, such as glucagon-like peptide 1 (GLP-1) receptor agonists (RAs), sodium-glucose co-transporter-2 (SGLT-2) inhibitors and dipeptidyl peptidase-4 (DPP-4) inhibitors, were used as add-on therapies secondary to metformin. In a recent meta-analysis, Fey et al. compared the effects of these compounds on cardiovascular outcomes in diabetic patients. They reported that compared with other antidiabetic drugs, SGLT-2 inhibitors are superior in reducing the cardiovascular mortality, hospitalization for heart failure and the appearance of renal damages, while the effects of DPP-4 inhibitors on cardiovascular and renal outcomes are comparable to placebo [139].

#### 6.2.2. Therapeutic Compounds to Reduce Formation of AGE

Data from literature clearly support the involvement of AGE products (from exogenous or endogenous sources) in the generation of micro- and macrovascular complications of diabetes. Notably, it was stated that AGE are possible mediators of the metabolic memory of cells in diabetes [5], their formation determining the evolution of diabetic complications even when glycemia is under strict control. Thus, the inhibition of AGE formation is one of the possible therapeutic approaches to reduce the diabetic burden.

Few therapeutic compounds were reported to block the formation of AGE, including metformin, derivates of vitamin B and statins. Metformin was reported to prevent AGE formation in vitro [140] and to reduce the levels of CML, while increasing plasma soluble RAGE in patients with metabolic syndrome [141].

Derivates of vitamin B6, such as pyridoxamine, were shown to be beneficial in reducing AGE levels and the vascular complications of diabetes. Recently, Pereira et al. reported that pyridoxamine significantly improved the function of EC in the aorta and mesenteric arteries of an animal model of non-obese T2DM by decreasing the vascular oxidative damage and AGE levels [142]. Using different animal models of diabetes, other studies showed that pyridoxamine can reduce the apoptosis of cardiomyoblasts in cardiac ischemia [143], impede the development of atherosclerotic lesions and improve the cardiac ejection fraction in diabetic mice with myocardial infarction [144], or reduce the arterial stiffening by inhibiting the glycation of aortic collagen [145]. Another derivate of vitamin B6, 2-aminomethyphenol pyridoxamine, was shown to reduce the CML levels, triglycerides and total cholesterol in the plasma of diabetic STZ-induced rats [146].

Lipid-lowering statins were constantly reported to inhibit the AGE/RAGE axis. Clinical trials indicate that atorvastatin, simvastatin, pravastatin and pitavastatin have potent AGE-lowering effects, independent of the glycemic control of the patients [147,148,149]. Several mechanisms to explain the AGE-lowering effects of statins have been described. First, it was shown that statins stimulate ADAM-10 mediated RAGE shedding, increasing sRAGE and the subsequent AGE clearance [150]. Since it is known that oxidative stress is an important participant in the formation of advanced glycoxidation and lipoxidation end products, it is reasonable to accept that the antioxidant properties of the statins decrease AGE. In addition, a third mechanism can be proposed, related to the lipid-lowering capacity of statins: the decrease in the cholesterol-rich LDL due to statins administration results in a reduced formation of gLDL. Besides reducing AGE, the pleiotropic effects of statin include antioxidant, anti-inflammatory, immunomodulatory, anti-proliferative and endothelial protective effects, supporting their use in preventing the CVD risk in diabetic patients [151]. Their beneficial effects were reported in several cohort studies and clinical trials. The Heart Protection Study (HPS) shows that administration of simvastatin substantially reduces the major vascular events in diabetic subjects [152]. Beneficial effects were observed also for atorvastatin in the Anglo-Scandinavian Cardiac Outcomes Trial-Lipid Lowering Arm (ASCOT-LLA) or the Collaborative Atorvastatin Diabetes Study (CARDS), both studies reporting a decreased risk of nonfatal myocardial infarction and fatal coronary heart disease (CHD) in hypertensive diabetic patients without a previous history of CHD (ASCOT-LLA) and a significant reduction of acute CVD events (including stroke) in T2DM patients with normal LDL cholesterol levels [153]. Recently, Ramos et al. reported that statin therapy significantly reduces the risk of CVD in older patients (>75 years) with T2DM [154].

#### 6.2.3. Antioxidants to Decrease CVD in Diabetes

Knowing the important involvement of ROS in diabetes, compounds with antioxidant properties such as vitamin C, E, alpha-lipoic acid, allopurinol and statins [155,156,157,158] were used to improve the health of diabetic patients.

The obtained data are contradictory: the results from HOPE (Heart Outcomes Prevention Evaluation) trial show that antioxidants, like vitamin E, have no effect on cardiovascular outcomes in DM patients [159], while a recent meta-analysis based on published data indicates that antioxidant vitamins (in particular vitamin E) have benefits, such as reduction of glycated hemoglobin levels, enhancing antioxidant capacity and improvement of EC function in non-obese T2DM patients, supporting their use in protecting against the complications of diabetes [155,156,160](Akbar S et al., 2011). In a very recent study, Baziar et al. showed that alpha lipoic acid (ALA) may decrease the CVD risk by reducing the level of oxidized LDL in the plasma of T2DM patients without affecting their lipid profiles or glycemic indices [157]. Altunina et al. showed that ALA reduced the systemic inflammation (measured as decreased CRP, IL-6 and TNF-α) in T2DM patients with a history of non-Q-myocardial infarction [161].

### 6.3. New Promising Therapies to Alleviate ECD in Diabetes

In diabetic-associated vascular diseases, LDL are modified (glycated and/or oxidized) and play a key role in accelerating the progression of atherosclerosis. Chronic hyperglycemia enhances glucose-induced LDL oxidation and/or glycation, increasing their pro-atherogenic properties [17,107] and promoting vascular injury. A correlation between arterial tissue AGE and circulating AGE-apoB, and the contribution of AGE-specific receptors (RAGE) in atheroma formation was reported [162]. Thus, inhibition of RAGE is a promising therapy to promote vascular healing in diabetes.

Together with reduction of AGEs, the alteration of miRNAs profiles in the plasma of diabetic subjects is indicated to be involved in the promotion of diabetic complications even under strict glycemic control [10], suggesting that miRNA-based therapies could be efficient in the reduction of CVD in diabetes.

In the next section, the progress of therapies based on RAGE inhibition, or on re-establishment of a miRNAs profile to reduce ECD and diabetes complications will be presented. Future therapeutic strategies such as reduction of Lp(a) or the use of CRISP/Cas technology to increase the anti-inflammatory AGE-receptor complexes will also be discussed.

#### 6.3.1. RAGE Inhibitors

A new class of compounds proposed to reduce the diabetic burden is the RAGE inhibitors. The fact that knocking out the RAGE gene in mice does not distress their health and does not seem to affect their growth suggests that RAGE inhibition could be a safe in vivo therapeutic approach [163]. Small-molecular inhibitors targeting either the extracellular ligand-binding site or, more recently, the intracellular signaling domain of RAGE have been developed [163]. TTP488, also known as PF-04494700 or azeliragon, is an orally bioavailable small-molecule inhibitor for the extracellular domain of RAGE. TTP488 inhibits the binding of multiple RAGE ligands, including AGEs, HMGB1, S100B and Aβ. Unfortunately, no data regarding the effects of TTP488 in diabetes exist, as the effects of TTP488 are documented mainly in the context of Alzheimer’s disease where preclinical studies show that TTP488 slows the cognitive decline at small concentrations [163]. Another extracellular RAGE inhibitor named FPS-ZMI did not cause toxic side effects in mice, even at doses as high as 500 mg/kg [164] and was demonstrated to have beneficial effects, such as reducing the cardiac hypertrophy and inflammation in cardiac tissues in a mouse model of heart failure [165]. A set of intracellular inhibitors of the cytoplasmatic domain of RAGE was identified by Manigrasso et al. [166]. It consists of 13 small molecules able to inhibit competitively the interaction between the cytoplasmic domain of RAGE and DIAPH1 [166]. These compounds exhibited anti-migratory effects in cultured SMC and anti-inflammatory effects in THP-1 macrophages exposed to CML-AGE [166]. In addition, a subset of these inhibitors was able to reduce the ischemia/reperfusion injury in the hearts of a streptozotocin-induced diabetic mouse [31,166].

AGE proteins induce dysfunction of EC along the vascular tree in diabetes. While AGE-receptor complexes act as scavengers for glycated proteins, AGE-RAGE/TLRs interaction induces intracellular signaling pathways, leading to increased oxidative and inflammatory stress. Therefore, genome-editing technology would be a promising tool to upregulate AGE-receptor complexes and down-regulate RAGE/TLRs in order to counteract the harmful effects of AGE-proteins in EC [167]. Due to its high efficacy, good repeatability, simple design and low cost, the CRISPR/Cas9 technology is successfully used to develop innovative organ-specific therapies for better management of diabetic-associated vascular diseases [168].

#### 6.3.2. MiRNA Based Therapies

The identification of epigenetic mechanisms that affect the function of EC in diabetes opened also the pathway for developing new promising therapeutic interventions. MiRNAs–based therapies are centered on two main approaches: the miRNA inhibitory therapy that aims to downregulate the aberrantly over-expressed miRNAs and the miRNA replacement therapy, which aims to restore the activity of beneficial miRNAs downregulated by pathologic conditions [10]. Different in vitro and in vivo studies indicate that miRNA-targeted therapies could be beneficial in alleviating micro- and macro-complications of diabetes. In vitro, it was reported, for example, that upregulation of miR-149-5p reduced ECD induced by high glucose-exposure by increasing the level of NO and the expression of eNOS, while decreasing the levels of ET-1, vWF and ICAM-1 [169]. In vivo, it was shown that the knockout of miR-21 alleviated the microvascular damage, inflammation, and cell apoptosis in the retina of db/db mice through the decrease in PPARα [170]. Icli et al. showed that delivery of miR-135a-3p inhibitors to wounds of diabetic db/db mice speeded the wound closure and angiogenesis and increased the thickness of the granulation tissue through the activation of p38 MAPK signaling in EC [171]. In addition, the delivery of miR-126 significantly improved neurological and cognitive functions in T2DM-stroke mice and induced the polarization of macrophages towards the anti-inflammatory M2 phenotype in the ischemic zone [172]. Although far from being used in clinical practice, the strategies that target the epigenetic mechanisms used in combination with standard anti-diabetic treatments might represent an additional opportunity to reduce the complications of diabetes.

#### 6.3.3. Inhibition of Glycated-Lp(a)

To date, no therapeutic approaches have been described to reduce the level of glycated Lp(a) in diabetic patients. A reasonable way to do this is to decrease the Lp(a) synthesis in parallel with the hypoglycemic therapies. Analysis of trials investigating evolocumab effects in CVD patients reported significant dose-related decreases in Lp(a) levels, in parallel with the decrease in LDL-C and apolipoprotein B levels [173]. In the last decade, an increased interest in antisense therapy has been noticed. This approach consists of inhibiting the synthesis of specific proteins by using complementary oligonucleotides that bind to their mRNA in the nucleus. Data from a clinical study that investigated an antisense oligonucleotide that selectively reduces the synthesis of apo(a) in the liver, and consequently Lp(a) plasma levels, also show a reduction in oxidized phospholipids on apo(a) and a reduced monocyte inflammatory activation [173]. Conjugation of antisense oligonucleotide with a GalNAc3 complex guides the drug to the hepatocytes via the asialoglycoprotein receptor, making it more potent than the parent antisense oligonucleotide. Thus, the necessary dose of the drug will be reduced and its tolerability improved [174].

## 7. Discussion

Diabetes is a complex disease, affecting numerous organs and the entire vascular tree. Accelerated atherosclerosis represents a major complication of the macro-vasculature of diabetic patients that can cause major acute cardiovascular events. Endothelial dysfunction has been demonstrated in both the peripheral and coronary arteries circulation. Alterations in lipid metabolism are at the core of T2DM phenotypes and probably greatly contribute to the increased risk of cardiovascular disease associated with diabetes. An important player in the induction and progression of atherosclerosis in diabetes is the interaction between the glycated and/or oxidized Lp and EC. In this context, more documented is the interaction between EC and gLDL or gHDL, while data regarding gLp(a) are scarce and require in-depth investigation. The mechanisms by which gLDL and gHDL induce ECD are complex and interconnected. Although gLDL can interact with different receptors, the harmful effects of gLDL on EC are induced especially by the interaction with RAGE, a process that triggers the activation of various signaling pathways and affects all EC functions, determining decreased NO production, the dysregulation of oxidants–antioxidants balance or alteration of the proper function of ER. The extended presence of gLDL in diabetes induces a pro-inflammatory status of EC and finally apoptosis. Notably, the combined exposure to high glucose concentrations exacerbates the effects of gLDL. Since the gLDL/RAGE interaction is the key step in generating ECD, the receptors for AGE-proteins are promising therapeutic targets to be addressed by novel gene-editing technology. These approaches could be complemented by the administration of molecules that restore the mitochondrial and ER functions in EC.

At the same time, glycation of HDL renders them dysfunctional, unable to exert anti-atherosclerotic actions due to the modified composition and activity of the associated enzymes and a decreased half-life in the plasma of diabetic patients. The gHDL not only lose their anti-atherogenic potential due to the glycation of its main proteins (apoAI, apoAII, apoE, PON-1) but become pro-atherogenic by enrichment with pro-oxidant and pro-inflammatory molecules, such as the pro-oxidant enzyme MPO and the pro-inflammatory protein SAA. However, many other issues regarding the effects of gHDL in EC (such as modulation of the intracellular antioxidant system or of the fibrinolytic system) and the precise mechanism by which gHDL affects the function of EC are still missing, so more studies are needed.

Certain compounds with anti-oxidant properties were effective to alleviate ECD in vitro. Alas, the administration of exogenous anti-oxidants to humans failed to improve the oxidative status and HDL function and its ensuing protective role in EC. The future employment of the gene-editing technology [CRISPR/(d)-CAS9] in vivo to upregulate endogenous anti-oxidant proteins, in particular those associated with HDL, could be a promising approach to improve EC function in diabetes. Taking into account the important impact of gLp on EC function, any new therapy that will be developed in the future to improve EC function in diabetes needs to be accompanied by a hypoglycemic and/or lipid-lowering therapy to ensure a less aggressive environment and to inhibit the formation of AGE-Lp.

Identification of LDL and HDL as important carriers of miRNAs with a dynamic profile under pathological conditions may result in the development of new therapies to improve EC function by delivery of specific protective miRNAs or inhibitors for the pro-atherogenic ones. However, the overall efficacy of these strategies needs to be further examined in pre-clinical and clinical trials.

Reversal of endothelial dysfunction is an open and exciting field of investigation, of fundamental relevance for many diseases, and in particular for diabetes and atherosclerosis. The progress of the biotechnology field will permit the use of targeted nanosystems such as nanoliposomes or nanoemulsions, or the specific regulation of different proteins based on the new CRISP/Cas9 technology to reverse endothelial dysfunction in diabetes, as well as other pathologies. In the future, these and other promising therapies focused on EC-based translational approaches will provide powerful tools to increase the quality of life of diabetic patients.
